# The Book World of Medicine and Science

**Published:** 1900-11-03

**Authors:** 


### The Book World of Medicine and Science.

The Principles and Practice of Hydrotherapy: A Guide to the Application of Water in Disease. For Students and Practitioners of Medicine. By Simon Baruch, M.D. With numerous illustrations. (London: Baillière, Tindall, and Cox. 1900. Price 16s. net.)

This work is stated to differ from all other works on hydrotherapy in that it is written by a general practitioner for the guidance of his colleagues; but we think that before the reader has gone far he will have arrived at the conclusion that although Dr. Baruch may call himself a general practitioner, which indeed his career and his connection with various branches of his profession show him to have been, he has made himself a sufficient master of the details of hydrotherapy to be considered and to rank as a specialist on that subject. The object of the book is not, it is stated, to write a panegyric upon the water cure, but to discuss water as a remedial agent, precisely as other medicinal agents are discussed in the text-books on therapeutics; and this object, as it seems to us, the author accomplishes with very considerable success, always, be it noted, with somewhat of a bias towards water. As Dr. Baruch very properly says, the idea that chronic disease must be sent away from home to obtain good results from hydrotherapy is fallacious, or at any rate would be fallacious if the general practitioner were able to give the same carefully thought-out advice and supervision as can be given by the physicians attached to large hydrotherapeutic establishments. A very considerable proportion of the most prevalent chronic diseases do not in fact require institutional treatment, but they do demand a careful adjustment of the baths, in regard to temperature, duration, and technique, to each individual case; and although, as things are, this is often to be obtained more readily at an institution than elsewhere, there is no reason why the requisite knowledge on this subject should not be acquired by the general practitioner as on any other department of therapeutics.

Dr. Baruch describes hydrotherapy as including the application of water in any form—from the solid and fluid to vapour, from ice to steam, internally and externally—but as not including the use of mineral waters when these depend upon their mineral constituents for their therapeutic efficacy, which, however, he says, "is rarely the case." The word "hydriatric," which he pretty constantly employs, he defines as a short and equally expressive substitute for the adjective hydrotherapeutic. On the whole it seems likely to be a useful addition to our vocabulary.

Commencing with chapters on the anatomy, physiology, and functions of the skin, the author goes on to deal with the rationale of the action of water upon the distribution and composition of blood, upon the respiration, the muscular system, and the temperature. As is only to be expected, pretty full theoretical reasons are given for all the results claimed. It is possible to obtain quotations from leading physiological works to illustrate if not to prove all kinds of hypotheses, and this is very thoroughly done by Dr. Baruch. There is chapter and verse for everything.

When we come to the practical portion of the work all is most admirable, more especially the descriptive parts, which deal with the methods which may be employed for the application of water and for obtaining its various effects.

These chapters are very full, and are well illustrated by an extensive series of photographs showing almost every stage of the processes described. It must not be imagined, however, that the book is a mere handbook of methods. Everywhere the author's opinions as to the indications for these baths and the views of other authorities on the subject are clearly and fully stated, and often in a very interesting manner. In passing we would here refer to what he says about the use of cold bathing in typhoid fever, and this more especially because there are many physicians who still seem to believe that bathing does but reduce temperature, while some seem to imagine that pyrexia can be as well combated by exposure to cold as by the use of baths. Dr. Baruch insists that the idea that high temperature is the chief determining cause of the fatality in typhoid fever must now be abandoned. This, of course, is by no means new, but it is useful along with the other point he makes—namely, that it is not by reducing temperature that cold bathing acts.

"It has been clearly demonstrated," he says, "by numerous trustworthy observers that the reflex stimulus aroused by the shock to the extensive peripheral nerve endings so energises the nerve centres which furnish innervation for circulation, respiration, digestion, tissue formation, and excretion that the system is enabled to tide over the dangers which would ensue from failure of these functions. This is in a nutshell the effect of cold bathing in infectious fevers; the simple cooling effect on the blood occupies a secondary though not unimportant office."

Although we must confess that we do not quite understand the word "energise," any more than we can attach any definite meaning to the term "vivifying" as applied a little later on to describe the effect of the cold bath, there is but little doubt that the idea which Dr. Baruch endeavours to express is correct, and that it is by some stimulating—again we drop into undefinable terms—influence, rather than by mere heat abstraction, that bathing produces its marvellous results. Dr. Baruch goes on to say: "Our English confrères have evidently not grasped Brand's idea, else their large material would have surely enabled them to see practical demonstrations of its enormous superiority over the expectant plan." The following remark about expectancy is not without its humour. The expectant plan, he says, "is correctly named, because the physician is constantly expecting some complications, to cope with which he rarely feels capable."

We have read the book with much interest. It contains a large amount of information on a series of important therapeutic measures which are almost entirely neglected in ordinary text-books, and it certainly helps to fill an important gap in medical teaching. Indeed, it is probably to the neglect of the teachers and the text-book writers to give definite directions as to the duration, the frequency, and the manner in which water is to be used in treatment that the failure of hydrotherapy to obtain full professional recognition is largely to be attributed.

